# Functional near-infrared spectroscopy-based prefrontal cortex oxygenation during working memory tasks in sickle cell disease

**DOI:** 10.1117/1.NPh.10.4.045004

**Published:** 2023-10-17

**Authors:** John Sunwoo, Payal Shah, Wanwara Thuptimdang, Maha Khaleel, Patjanaporn Chalacheva, Roberta M. Kato, Thomas D. Coates, Michael C. K. Khoo

**Affiliations:** a University of Southern California, Department of Biomedical Engineering, Los Angeles, California, United States; b Massachusetts General Hospital, Harvard Medical School, Athinoula A. Martinos Center for Biomedical Imaging, Boston, Massachusetts, United States; c Children’s Hospital Los Angeles, Keck School of Medicine, University of Southern California, Hematology Section of Children’s Center for Cancer, Blood Disease and Bone Marrow Transplantation, Los Angeles, California, United States; d Prince of Songkla University, Faculty of Medicine, Institute of Biomedical Engineering, Department of Biomedical Sciences and Biomedical Engineering, Hat Yai, Songkhla, Thailand; e Carnegie Mellon University, Department of Biomedical Engineering, Pittsburgh, Pennsylvania, United States; f Children’s Hospital Los Angeles, Keck School of Medicine, University of Southern California, Division of Pediatric Pulmonology, Los Angeles, California, United States

**Keywords:** sickle cell disease, cognitive decline, *N*-back working memory tasks, functional near-infrared spectroscopy, prefrontal cortex, stroke

## Abstract

**Significance:**

Sickle cell disease (SCD), characterized by painful vaso-occlusive crises, is associated with cognitive decline. However, objective quantification of cognitive decline in SCD remains a challenge, and the associated hemodynamics are unknown.

**Aim:**

To address this, we utilized functional near-infrared spectroscopy (fNIRS) to measure prefrontal cortex (PFC) oxygenation responses to N-back working memory tasks in SCD patients and compared them with healthy controls.

**Approach:**

We quantified the PFC oxygenation rate as an index of cognitive activity in each group and compared them. In half of the participants, a Stroop test was administered before they started N-back to elevate their baseline stress level.

**Results:**

In SCD compared to healthy controls, we found that (1) under a high baseline stress level, there were significantly greater oxygenation responses during the 2-back task, further elevated with histories of stroke; (2) there was a marginally slower N-back response time, and it was even slower with a history of stroke; and (3) the task accuracy was not different.

**Conclusions:**

Additional requirements for processing time, PFC resources, and PFC oxygenation in SCD patients offer an important basis for understanding their cognitive decline and highlight the potential of fNIRS for evaluating cognitive functions.

## 1 Introduction

Sickle cell disease (SCD) is an inherited genetic blood disorder affecting ∼1 in 500 individuals of African descent in the United States. SCD is characterized by painful, frequent vaso-occlusive episodes due to microcirculatory occlusions caused by rigid, sickle-shaped red blood cells. The vaso-occlusive episodes accompany severe pain crises, originating from the surrounding tissue damage due to oxygen deprivation. One common treatment is blood transfusions, which require costly hospital visits and follow-ups. Unfortunately, many SCD patients, due to their disadvantaged socioeconomic status, often cannot access this essential care. This leads to poorer health outcomes and a diminished quality of life compared with patients with other chronic diseases in the United States.

In recent years, numerous studies have aimed at noninvasively identifying physiological markers of SCD severity, with the goal of guiding longitudinal treatments. Most studies focused on the alteration of sensory function and the autonomic nervous system caused by frequent pain crises. Some explored the neuropathways and transmitters responsible for elevated pain sensitivity in SCD.^1^^–^^3^ Other studies identified an elevated, neurally mediated peripheral vasoconstriction response to stimuli that trigger pain crises, such as heat or cold.^4^^–^^7^ These findings were further demonstrated by altered responses in SCD’s autonomic nervous system during a head-up-tilt test,^8^ and additional studies have explored similar markers.^9^^–^^12^

Cognitive decline is another complication of SCD, but its dynamics are not fully understood. It is known that cerebral microcirculatory occlusions can cause silent or overt strokes, which can lead to cognitive decline.^13^^–^^15^ Previous studies have shown a correlation between intelligence quotient scores and MRI-based stroke severity assessments in SCD;^16^ children with SCD have exhibited difficulties in performing specific memory tasks;^17^ and recent MRI studies have revealed shrinkage or hyperintensity of the white matter (indicating infarcts) in SCD, suggesting evidence of, or factors contributing to, cognitive decline.^18^^,^^19^ However, due to feasibility reasons, these studies rely on MRI-based, steady-state structural brain imaging rather than observing real-time brain activations during cognitive tasks. Consequently, our understanding of cognitive decline in SCD—based on brain hemodynamics—is still lacking.

To address this gap, we conducted a study on functional brain activity in SCD using wearable sensors that can be easily deployed and provide objective signal features of cognitive brain activity. We hypothesized that neurovasculopathy, caused by chronically inadequate cerebral blood flow as in SCD, leads to poor and slow cognitive functions. We further hypothesized that these functional deficits would be reflected in the prefrontal cortex (PFC) oxygenation measured using functional near-infrared spectroscopy (fNIRS) during experimentally designed cognitive tasks.

We administrated N-back tasks, which require short-term working memory, with simultaneous fNIRS monitoring in 23 SCD patients and 18 age- and race-matched healthy subjects. N-back memory tasks are commonly used to assess mental workload as they activate the PFC, which is responsible for cognitive processing.^20^ We also utilized fNIRS as it is portable and convenient for *in situ* experiments. Previous fNIRS-based studies have found increased brain oxygenation within the PFC during demanding memory tasks in non-SCD participants.^21^^–^^25^ Although MRI-based studies on resting state in SCD have been reported,^18^^,^^26^^,^^27^ to date, we are not aware of any studies that utilized fNIRS to characterize PFC oxygenation responses to a cognitive task in SCD.

## 2 Methods

### 2.1 Subject Demographics

This study was approved by the Institutional Review Board and conducted at Children’s Hospital Los Angeles (CHLA). We enrolled 23 patients with SCD who received care at CHLA and 18 age- and race-matched control subjects. Participants had to be older than 11 years and free of vaso-occlusive crises or hospitalization within the past 10 days and not have an anxiety disorder. All participants provided written consent or assent before the study. Within the SCD group, we further identified eight patients with a history of stroke (SCDhs1). Detailed demographics are shown in Table 1.

**Table 1:** Population characteristics.

	Healthy control N=18	SCD N=23	^a^P-value
Age (years)	19 ± 5	21 ± 6	0.4
Female	8 (44)	10 (43)	1.0
Hemoglobin (g/dL)	12.7 (12.0 to 14.5)	9.6 (8.6 to 11.7)	<0.001
^b^Hemoglobin deficit (rg/dL)	0.0 (0.0 to 0.2)	3.2 (1.3 to 4.5)	<0.001
Treatment			
Chronic blood transfusion		9 (39)	
Hydroxyurea		12 (52)	
History of stroke			
^c^Silent		5 (63)	
Overt		^d^3 (37)	

Note: Data are presented as N (column %) for categorical variables, except when presented as mean ± SD for normally distributed continuous variables and when presented as median (25 to 75th) for non-normal continuous variables.

a T-test was used for normally distributed continuous variables, Wilcoxon/Kruskal–Wallis Rank Sum test was used for non-normal continuous variables, and Fisher’s exact test was used for categorical variables.

b Normal hemoglobin count was computed using the subject’s age and sex corresponding to “normal hemoglobin count ranges widely accepted by physicians” from the Disabled World hemoglobin level chart.^28^

c Being a “silent” stroke was determined clinically and from patients’ MRI images and the signs of cognitive decline assessed by neuropsychological testing.^29^

d Locations of stroke. Patient #1: bilateral deep white matter front lobe, bilateral centrum semiovale, and bilateral cerebellar lesions; patient #2: bilateral centrum semiovale and right frontal white matter infarctions; patient #3: the stroke was resolved at the time of the study, and the original locations of stroke (occurred 18 years prior to the study date) are unknown because of no electronic medical record.

### 2.2 Study Design

**Fig. 1:**
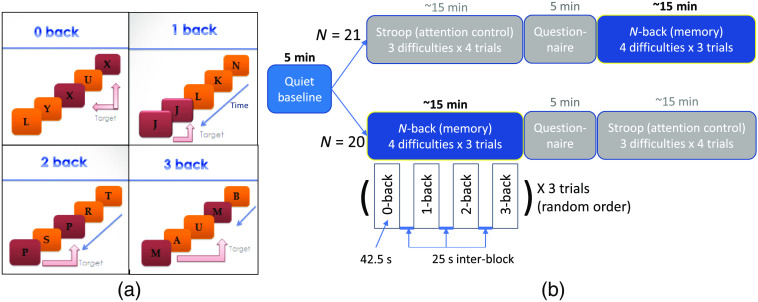
N-back working memory task, while half engaged in a word-color-incongruent Stroop task prior to N-back. (a) The N-back test required a subject to remember the sequence of letters presented on the screen and then press a button when the currently showing letter is the target. (b) Each N-back block with the same difficulty level was presented three times, for a total of 12 N-back blocks. A Stroop test was also assigned in half of the subjects before the N-back test to elevate their baseline stress level. PFC responses and behavioral scores during Stroop were not analyzed in this work.

We utilized the N-back working memory task to evoke prefrontal oxygenation during cognitive workload.^20^^,^^30^^,^^31^ However, not all participants started with the same baseline. Although half of the subjects began directly with the N-back task, the other half engaged in a word-color-incongruent Stroop task, a different type of cognitive workload that potentially elevated their baseline stress levels. This Stroop task and its induction of mental stress have been published by our group.^32^ Between the Stroop and N-back tasks, we incorporated an ∼5-min questionnaire about the subjects’ state-trait anxiety levels [Fig. 1(b)].

We began by measuring the subjects’ PFC oxygenation for 5 min without any task. Following this period, we presented N-back tasks on a computer screen via E-prime 2.0 software (Psychology Software Tools, Inc.). In a single block of N-back tasks, a sequence of alphabetic letters was presented for 0.5 s, followed by a 2.5-s blank screen, and participants were instructed to press a keyboard button when the current letter matched the one presented N-backs ago (N=1, 2, or 3). For the 0-back condition, the target letter was “X” [Fig. 1(a)]. Each N-back block lasted 42.5 s and had 4 to 6 correct targets for a button press. Each level (0- to 3-back) was presented by the N-back block, three times in random order, resulting in a total of 12 N-back blocks per participant (4 levels × 3 trials). A 25-s rest period followed each block [Fig. 1(b)]. The E-prime software recorded the response time (in ms resolution) and the accuracy of the participant’s performance. For each N-back block, data were excluded from the analysis if the participant did not provide any keypress responses.

### 2.3 Noninvasive Brain Oxygenation and Physiological Measurements

**Fig. 2:**
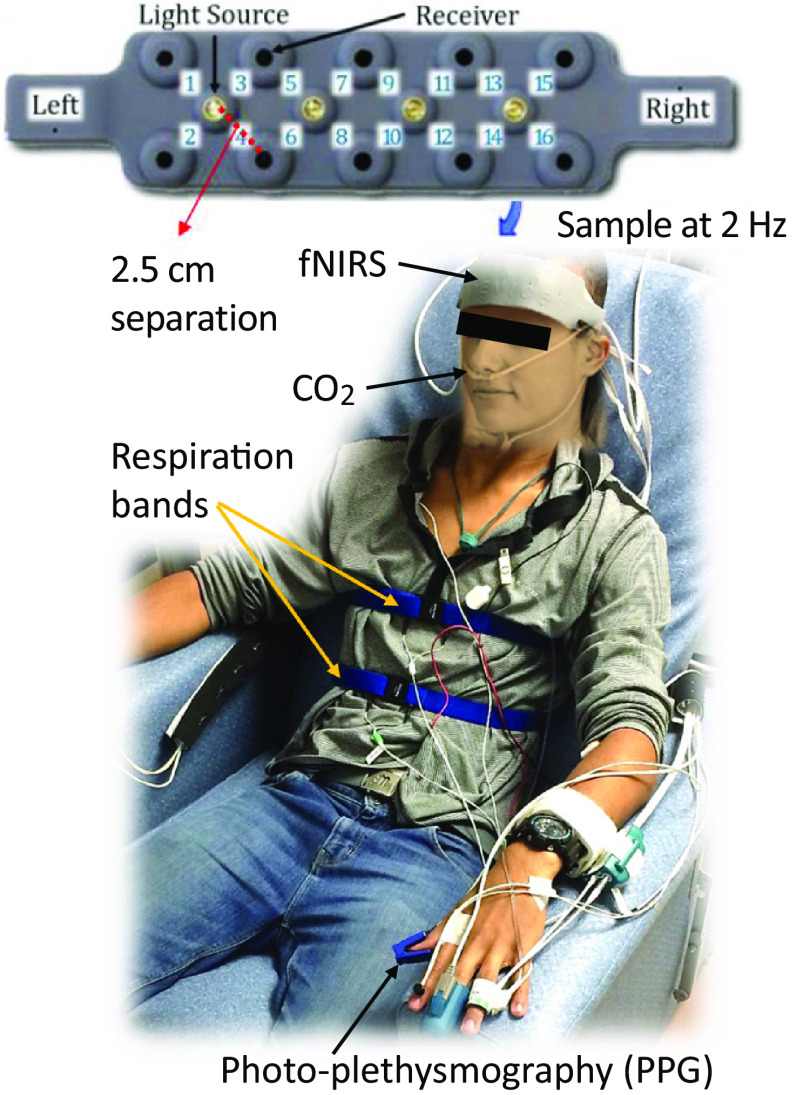
PFC and peripheral responses to mental task were made using fNIRS, capnography, photo-plethysmography (PPG), and respiration bands.

We recorded the changes in PFC hemodynamics using fNIRS (Biopac Systems Inc.) concurrently with peripheral physiology, including fingertip blood volume change using photoplethysmography (PPG, Nonin Medical Inc.), end-tidal ${\mathrm{CO}}_{2}$ (ETCO2) through a nasal cannula (Vacumetrics Inc.), and respiratory patterns using inductive belts around the chest and abdomen (Pro-Tech zRIP DuraBelt), as illustrated in Fig. 2. The fNIRS was operated at 785 and 850 nm wavelengths, as well as with an “off” period to collect and account for ambient light interference. The two wavelengths of light and dark periods were time-multiplexed across four LED sources, surrounded by the detectors at 2.5 cm separation. This setup provided a data rate of 2 Hz for each of the 16 fNIRS channels on the forehead. More technical details about this fNIRS device can be found in previous publications.^22^^,^^33^^,^^34^ PPG and ETCO2 (or respiration in two subjects without ETCO2) measurements were acquired to account for variations in participants’ autonomic nervous system and ventilation as they can influence the hemodynamics of extracerebral layers and non-neuronal components, potentially introducing bias to fNIRS measurements.^31^^,^^33^^,^^35^^,^^36^ PPG amplitude can indicate vasoconstriction responses in extracerebral layers, and ETCO2 or respiration offers a noninvasive measure of arterial PCO2, known to profoundly impact cerebral blood flow and consequently, fNIRS measurements. All of the aforementioned physiological signals, along with “begin” and “end” markers from the fNIRS device and N-back software (for data synchronization), were recorded on a Biopac MP 150 system at a 1 kHz sampling rate (Biopac Systems Inc.).

### 2.4 Signal Preprocessing to Remove Non-Neuronal Influences

#### 2.4.1 Motion artifact identification and correction

We used custom MATLAB scripts (The Mathworks Inc.) for signal processing and analysis. Motion artifacts were identified and corrected using a combination of spline and wavelet filtering, which has been recommended in recent review papers.^33^^,^^37^^,^^38^ These techniques were available as MATLAB functions in the open-source Homer 3 v1.58.0 software package. We made a few modifications to their thresholds and settings to suit our data rate at 2 Hz.^39^^–^^41^

First, we subtracted ambient light from the measured light intensity at 785 and 850 nm wavelengths. We then removed individual fNIRS channels with a saturated, low, or poor signal-to-noise ratio (SNR) assessed by [average signal intensity]/[standard deviation], as implemented in the “hmrR_PruneChannels.m” function from the Homer software, with “dRange” set to [40, 4000] and “SNRthresh” set to 2. Next, we estimated the changes in PFC oxygenated and deoxygenated hemoglobin concentration (ΔHbO and ΔHbR in uM) by applying modified Beer’s law on the light intensity measurements.^42^

Motion artifact suspects were identified using the “hmrR_MotionArtifactByChannel.m” function using a 2-s time window (“tMotion” = 2) with an additional ±1  s around the identified motion artifact period (“tMask” = 1), if the signal exceeded 30 times the running standard deviation (“STDEVthresh”) or had an instant change in signal amplitude >0.8  uM (“AMPthresh”). These settings were adapted from Ref. 39 and adjusted for our device’s sampling rate.

We corrected motion artifact periods, found individually per channel, using spline filtering (“hmrR_MotionCorrectSpline.m”), which involved fitting a cubic spline to the motion artifacts, subtracting the fit from the original signal, and adjusting the baseline to make the signal continuous around the corrected period. We used the default options suggested by Homer software, except we increased the windowing size for computing the signal mean applied in baseline shifting to “dtShort” = 1.5 and “dtLong” = 15 s to accommodate our 0.5-s data rate. Next, the spline-filtered signals were filtered using a wavelet transformation-based method, “hmrR_MotionCorrectWavelet,” which found the distribution of wavelet coefficients, rejected the outlier coefficients at the tails (using “iqr” = 1.5, meaning 1.5 times the IQR, as suggested by the Homer software), and reconstructed the original signal.

#### 2.4.2 Model-based filtering (MBF) for reducing extracerebral and respiratory influences

**Fig. 3:**
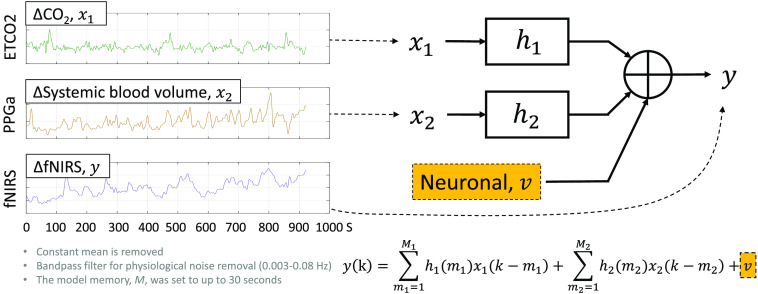
Two-input linear dynamic modeling for explaining the contributions from ${\mathrm{CO}}_{2}$ and skin blood flow in original fNIRS measurements. Impulse responses ($h_{1}$ and $h_{2}$) are found using Laguerre-basis function expansion and least squares methods.

To reduce the extracerebral and respiratory influences in original fNIRS measurements, we used a linear, time-invariant signals and systems model to fit non-neuronal confounders and subtract them from the original signal for each fNIRS channel (Fig. 3). Specifically, we employed a 2-input 1-output model to estimate the impulse responses between confounders and fNIRS measurements. We utilized a linear combination of Laguerre basis functions to estimate the impulse responses, minimizing the number of unknown parameters (2 to 6 basis functions and up to 30 s of memory).^43^ The Laguerre basis function expansion technique has been effective in modeling biological systems, including neuron models.^44^ To prevent overfitting, we applied the message-description-length technique,^45^ which penalizes using too many Laguerre basis functions while minimizing the variance of the residual.^46^

The first confounder that we targeted was the hemodynamics from the superficial layer (mainly the scalp), estimated using the fingertip PPG beat-to-beat amplitude (PPGa).^5^^,^^10^ The second confounder was the changes in cerebral hemodynamics due to ${\mathrm{CO}}_{2}$ fluctuation resulting from varying breathing patterns,^35^^,^^47^ which was reflected in ETCO2. A band-pass filter at 0.003 to 0.08 Hz was applied to remove slow signal drift and other oscillatory physiological noise outside the expected N-back oxygenation responses.

As a result, we found dynamic relationships (i.e., impulse responses; h1 and h2 in Fig. 3.) between the non-neuronal confounders and the fNIRS measurements that best explained the influences of ${\mathrm{CO}}_{2}$ and extracerebral blood flow in the original fNIRS measurement. We fitted the PPGa and ETCO2 signals into ΔHbO and ΔHbR signals, and we considered the residual ΔHbO and ΔHbR signals, v in Fig. 3, as refined time series in response to neurocognitive activity.^31^ This process was performed for each channel in each subject.

#### 2.4.3 Correlation-based signal improvement as a final refinement step

To further enhance neurally evoked oxygenation responses to N-back, we applied the correlation-based signal improvement (CBSI) method to the residual ΔHbO and ΔHbR signals, v, from the previous model-based filtering. The CBSI method has proven effective in removing non-neuronal signal artifacts as it assumes that neurally activated HbO and HbR signals are anti-correlated, whereas motion artifacts increase their positive correlation.^48^^,^^49^ Another advantage of the CBSI method was to reduce the signal dimensionality, from having original HbO and HbR to either CBSI-HbO or CBSI-HbR as they become mirror images of each other due to the algorithm. The processing parameters for CBSI remained the same as the original method published.^48^ Finally, as described in the subsequent section, we quantified PFC oxygenation responses using the CBSI-enhanced residual ΔHbO signal, which is referred to as “oxygenation” time series throughout the rest of this paper.

### 2.5 Response Quantification and Analysis

#### 2.5.1 Total oxygenation change

**Fig. 4:**
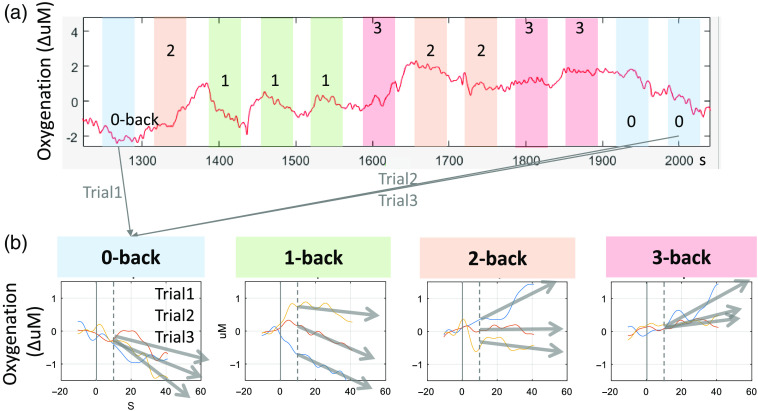
Quantification of the PFC oxygenation responses using the slope of the fNIRS signal measured during an N-back task. (a) The time course of PFC oxygenation changes due to different randomized N-back tasks obtained from one fNIRS channel. N-back tasks are grouped by difficulty and highlighted in the same color. (b) The response quantification using the linear line or the slope fitted over the oxygenation change.

Using the CBSI-enhanced residual ΔHbO signal, we quantified the PFC oxygenation responses by finding the slope of oxygenation during each trial period as an indicator of cognitive activation. To do so, a straight line was fitted on to the data in each trial window using “polyfit” in MATLAB. And as suggested in previous reports,^22^^,^^50^ positive slopes were interpreted as a constant recruitment of the PFC resources to solve difficult tasks, whereas more negative slopes were interpreted as less cognition activation occurring for easy tasks. We excluded the first 10-s period from each trial onset as it contained transient or unstable hemodynamics.^50^ Any trial that included the motion artifact periods exceeding 40% of the trial period including 5 s prior to the trial onset was excluded from the analysis, which was identified using the “hmrR_MotionArtifactByChannel.m” function in the previous step. Figure 4 shows one representative example of oxygenation time series during N-back tasks and how they were quantified into slopes. As a result, each subject produced a total of 192 PFC oxygenation slope responses (16 fNIRS channels × 4 levels of difficulty × 3 trials). Throughout this paper, this oxygenation slope serves as an index of cognitive activity and is interchangeably referred to as the “oxygenation rate” measured in ΔuM/s.

#### 2.5.2 Statistical analysis

We used a mixed model to investigate the effects of SCD and stroke history, N-back difficulty, and their interactions across four PFC quadrants (or referred to as “quads”) while accounting for the repeated measures from multiple N-back trials and four channels within each quadrant. Grouping by quadrant improved the signal-to-noise ratio while maintaining the spatial sensitivity. Our outcome variables included accuracy, response time, and slope of oxygenation during each N-back difficulty level (refer to Table 2). We also tested for possible confounding and interactions due to age, sex, and the “elevated stress” induced by Stroop. To ensure the normality of variables, we used the Shapiro–Wilk test and QQ-plots for each level of task difficulties and diagnosis groups and applied necessary transformations to make them more normally distributed. We also evaluated the residuals of the model for normality. Given the exploratory nature of this study, because there is no prior knowledge or data about PFC oxygenation response to N-back in SCD, we did not adjust for multiple comparisons but set an α level to 0.01 to indicate meaningful significance when assessing the main effects and the α level to 0.05 for covariates and interaction tests. Where appropriate, we computed the effect size using partial eta squared ($\eta_{p}^{2}=\frac{F\text{statistic}\times \text{factor degrees of freedom}}{\sqrt{F\;\text{statistic}\times \text{factor degrees of freedom}+\text{error degrees of freedom}}}$, with bench marks of small = 0.01, medium = 0.06, and large = 0.14) for multifactor analysis of variance (ANOVA), or Cohen’s d (derived by $\frac{t\;\text{statistic}}{\sqrt{\text{degrees of freedom}}}$, with bench marks of small = 0.2, medium = 0.5, and large = 0.8) for pair-wise comparisons.^22^^,^^51^^–^^53^ All analyses were performed using JMP Pro v15 Software (SAS Institute Inc.).

**Table 2:** Two-way repeated measures of ANOVA using a mixed model.

Responses	Within-subject factors^a^	Between-subject factors	Covariates
• Accuracy • Response time	N-back difficulty level	Diagnosis (SCD and SCD with stroke history)	• Age and sex • Elevated stress
Slope of oxygenation	• N-back difficulty level • PFC locations in four quadrants		

a To account for the repeated measurements in the within-subject factors (i.e., three repeated trials per difficulty level and four fNIRS channels per quadrant), each of the within-subject factors was set as a “random” effect in the mixed model.

## 3 Results

In this section, we present: (1) the effect of signal refining, including model-based filtering, (2) comparisons of task accuracy and response times, (3) oxygenation time series across all subjects, and (4) oxygenation rates after accounting for the elevated stress effect. Detailed figures that demonstrate (1) the effect of signal cleaning and (2) initial comparison of PFC oxygenation rates in quadrants before accounting for elevated stress are given in the Supplementary Material.

### 3.1 Signal Cleaning and MBF Results

**Fig. 5:**
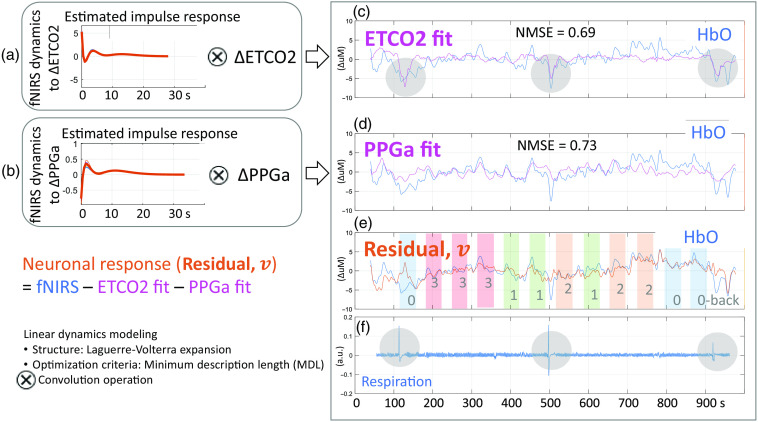
Example of MBF in one fNIRS channel, showing the ${\mathrm{ETCO}}_{2}$ fit on vasoconstriction responses to sighs and the PPGa fit on the rest of the background fluctuation. This fNIRS HbO change during the whole N-back session showed about 30% contribution from non-neuronal components, attributable to breathing (${\mathrm{ETCO}}_{2}$) and skin blood flow (PPGa). (a), (b) The estimated impulse responses associated with ${\mathrm{ETCO}}_{2}$ and PPGa, and (c), (d) their contributions to fNIRS HbO in terms of normalized mean square error (NMSE) = 0.69 and 0.73, respectively. (c), (f) The first row (in pink) highlights the contribution of ${\mathrm{CO}}_{2}$ to the original fNIRS signal, particularly notable during sigh events. (d) The second row shows the systemic/peripheral blood flow indicated by PPGa fit, embedded in the original fNIRS signal. (e) The fitted signal (non-neuronal influences) were subtracted so that they were mitigated in the final residual fNIRS signal, v. This process was done for each fNIRS channel.

Motion artifact detection based on the sliding window signal variation method, followed by spline and wavelet transformation filtering, removed both step and spike types of signal artifacts and improved the quality of the fNIRS data (Fig. S5 in the Supplementary Material). Subsequently, the model-based filtering corrected the potential signal bias caused by non-neuronal changes during the N-back task. Figure 5 illustrates an example of the MBF process, with the fitted impulse responses of the ETCO2 and PPGa on the first two rows [Figs. 5(a) and 5(b)]. On the right side of the first and the last panels, the pink line represents the fitted contribution of ${\mathrm{CO}}_{2}$ in the original HbO NIRS data, which showed a better fit/contribution when a sigh was present [Figs. 5(c) and 5(f), evident at time points around 100, 500, and 900 s]. Figure 5(d) shows the fit of PPGa changes onto the remaining fluctuation of the original fNIRS signal. Finally, the residual v was obtained by subtracting the fitted, non-neuronal contribution from the original fNIRS signal and used for subsequent analyses.

### 3.2 N-back Task Accuracy and Response Time

**Fig. 6:**
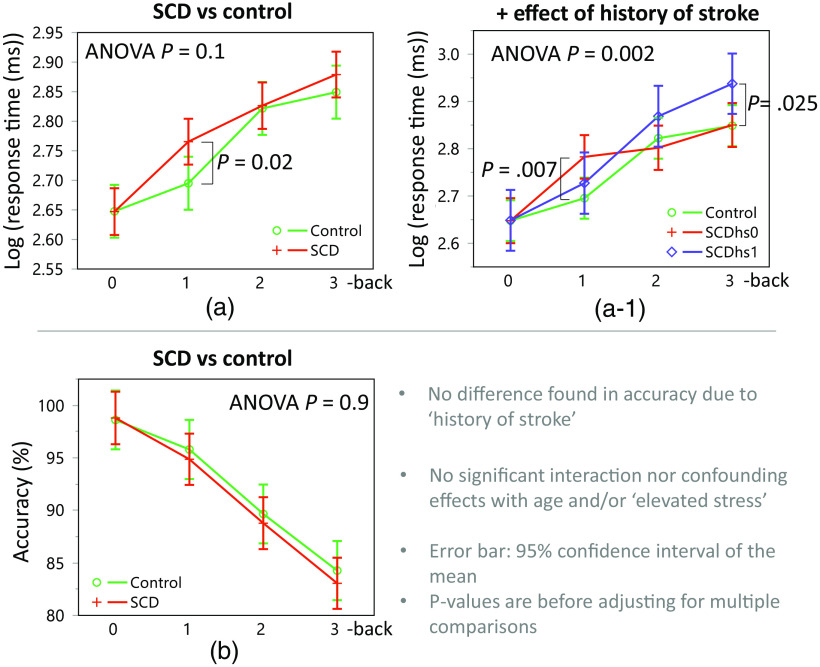
(a), (a1) Slower response times to N-back in the SCD groups than the healthy control group. (b) However, task accuracy was not different among the groups, suggesting that longer and less efficient processing is needed in SCD to complete a task as competently as in healthy controls. P-values shown are before adjusting for multiple comparisons, and the error bars indicate 95% confidence interval of the mean after accounting for repeated measurements in each subject. *SCDhs0/1: SCD without/with histories of stroke.

We observed significantly decreased accuracy and increased response time in response to more difficult N-back (P<0.0001, $F_{3,113.3}=72.08$, and effect size $(\eta_{p}^{2})=0.66$ for accuracy; P<0.0001, $F_{3,114.1}=77.94$, and effect size $(\eta_{p}^{2})=0.67$ for response times; see Fig. 6). Meanwhile, we did not find significant differences in accuracy related to SCD or history of stroke (ANOVA P=0.9 for each effect). For the response time, we applied a log-transform to achieve normality and found strong tendencies toward slower response times in the SCD group and SCD without the history of stroke (SCDhs0) during 1-back, as well as in the SCDhs1 group during 3-back, compared to controls (P=0.02, 0.007, and 0.025; $t_{91.53}=2.29$, $t_{82.42}=2.77$, and $t_{82.50}=2.11$; Cohen’s d=0.24, 0.31, and 0.23, respectively, without adjusting for multiple comparisons [see Figs. 6(a) and 6(a-1)]). There were no significant confounding or interacting covariates, including the elevated stress effect, associated with accuracy and response time.

### 3.3 Grand Average Responses to N-back in all Subjects

**Fig. 7:**
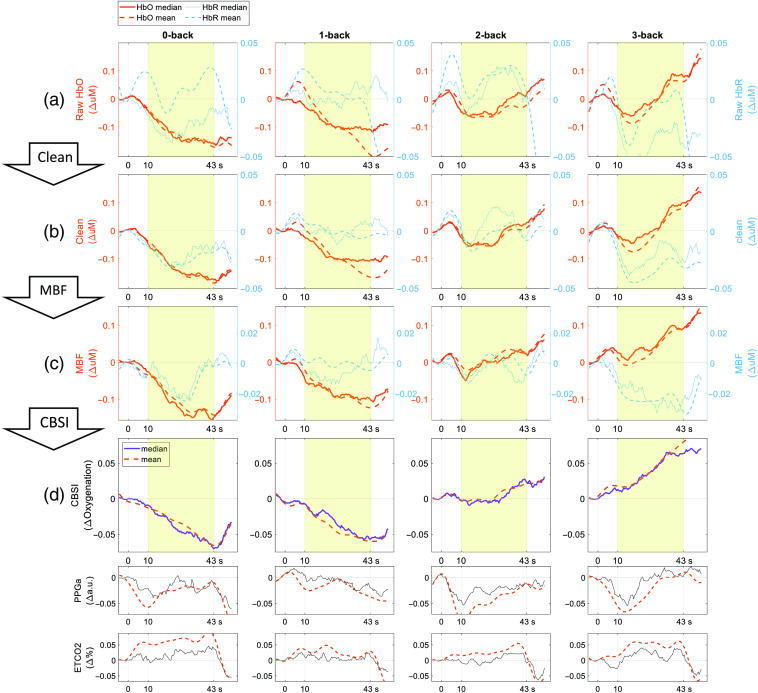
Effect of cleaning and improvement on N-back responses to become more characteristic in all 41 subjects. (a), (b) Motion detection algorithm and spline + wavelet filtering cleaned the signal. (b), (c) The proposed model-based filtering helped to reveal more positive and greater brain oxygenation changes as task difficulty increased, and they did not adversely alter the original signal shape. (c), (d) CBSI helped extracted neuronal activation by amplifying anti-correlation of HbO and HbR. CBSI-based signal was used as a “final oxygenation time series” for the analysis. Oxygenation responses 10 s after the trial onset (highlighted on the plots) were used to quantify the slope of the oxygenation response. The 10-s post-trial showed an oscillating trend returning to the baseline.

Figure 7 presents the average fNIRS time series from all 41 participants. Both the HbO and HbR responses showed a typical response trend to N-back, as indicated by the gradual increase or decrease in both the mean levels and slopes. And 10-s post-trial showed oscillating trends returning to the baseline. The motion detection algorithm and spline + wavelet filtering reduced the signal variability, bringing the median and mean closer together [Figs. 7(a) and 7(b)]. The effect of the MBF can be seen from Figs. 7(b) and 7(c), especially evident in the 3-back condition, which shows less contamination from the peripheral blood flow as indicated by PPGa. The last two rows of Fig. 7 show larger fluctuation and vasoconstriction responses reflected in the PPGa during the first 10 s of each N-back task, and mild and sustained increase in ETCO2 trends were observed during the tasks. The following CBSI process further isolated the oxygenation increase based on the expected anti-correlation between HbO and HbR during neural activation. After each signal refining step, we obtained more typical N-back responses, characterized by increasing HbO or decreasing HbR signal mean levels and slopes with more difficult N-back and by less signal fluctuation during the 10-s transient period.

### 3.4 Indications of Increased PFC Oxygenation in SCD Compared to Healthy Controls

**Fig. 8:**
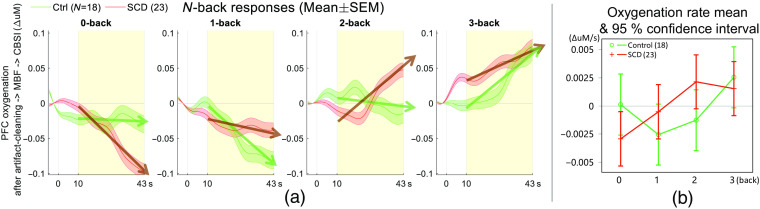
Comparison of average PFC oxygenation responses to N-back tasks of varying difficulty in control and SCD groups. An upward trend of PFC oxygenation in response to difficult tasks (i.e., 2 and 3 backs) is observed across both groups. (a) The PFC oxygenation response is quantified as the slope of the linear fit from 10 s post the onset of each trial (highlighted in yellow). This quantification reveals a marked increase in PFC oxygenation during 1- and 2-back tasks in the SCD group as compared to the control group. (b) The mean and 95% confidence interval of the whole PFC oxygenation over the N-back tasks are presented. Although no significant contrast was discovered at alpha = 0.01, marginal differences were observed, with $P_{2\text{-back}}=0.06$.

Figure 8 presents average PFC oxygenation responses from SCD and healthy controls. Both groups displayed flat or negative oxygenation during the 0- and 1-back tasks, while showing increased oxygenation during the 2- and 3-back tasks. Notably, a marked contrast in oxygenation slope during the 2-back task suggested hyperactivation in the SCD group. However, this did not reach the statistical significance at α level 0.01. Furthermore, the control group did not exhibit the expected negative oxygenation changes during the 0-back task, which prompted further investigations as discussed in the next sections.

Next, we grouped the oxygenation slope for each channel into PFC quadrants (i.e., treated as four repeated measures). Initially from this quadrant analysis, we found potentially greater oxygenation in the SCD group during the 2-back task compared to the healthy control group at quad 3, although this difference did not reach our threshold for statistical significance (P=0.017, $t_{226.4}=2.408$, d=0.16, see Fig. S3 in the Supplementary Material). However, this finding was accompanied by a significant interaction with the elevated stress factor (i.e., whether the subject completed the N-back or the Stroop test first; $P_{\text{stress}*\text{difficulty}}=0.012$, $F_{3,111.2}=3.80$, $\eta_{p}^{2}=0.09$, and $P_{\text{stress}*\text{difficulty}*\text{diagnosis}}=0.029$, $F_{3,111.2}=3.13$, and $\eta_{p}^{2}=0.08$).

### 3.5 Interaction with Elevated Stress

**Fig. 9:**
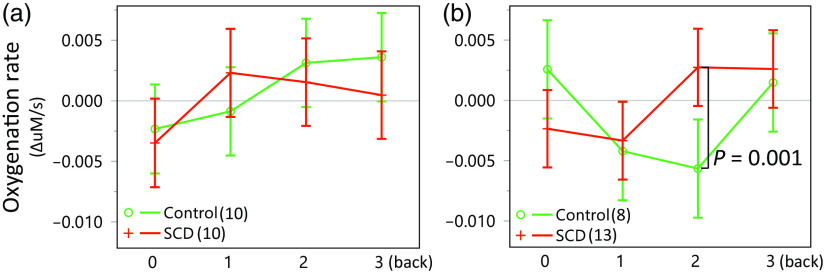
Interactions due to Stroop mental stressor prior to the N-back task, with the control group showing a typical N-back response trend in the (a) “N-back first” group, and (b) the “elevated stress” group showing a positive 0-back response and suppressed oxygenation during 2-back (P=0.06 and 0.001, respectively).

To further investigate the interaction by the elevated stress, we stratified the subjects into those who completed the Stroop test first (elevated stress group; 21 subjects) and those who completed the N-back test first (minimal stress group; 20 subjects). As a result, as indicated in Fig. 9, the minimal stress group displayed no significant difference or atypical trends [Fig. 9(a)]. However, the elevated stress subjects, particularly in the control group, exhibited unanticipated positive oxygenation trends during 0-back and more negative oxygenation trends toward the 2-back task [i.e., not monotonically increased, Fig. 9(b)]. And when we normalized the responses to 0-back tasks, these unanticipated trends in the control group made the N-back PFC oxygenation in SCD significantly higher than the control group (see Supplementary Material).

In the elevated stress group, the SCD group showed greater oxygenation changes during 2-back compared to the control group. Across all quadrants, the differences were statistically significant (P=0.001, $t_{142.5}=3.23$, and d=0.27), whereas the healthy control group showed positive and marginally greater oxygenation than the SCD group during 0-back (P=0.06, $t_{143}=-1.89$, and d=0.16). Further, *post hoc* analyses revealed that quads 1 to 3 had significantly higher oxygenation in the SCD group during 2-back (P=0.01, 0.008, and 0.0004, respectively; $t_{104}=2.60$, d=0.25 for quad 1, $t_{101.2}=2.72$, d=0.27 for quad 2, and $t_{101.2}=3.69$, d=0.37 for quad 3; not shown). Conversely, quad 2 suggested elevated oxygenation in healthy controls during 0-back (P=0.016, $t_{101.8}=-2.45$, and d=0.24). Meanwhile, in the minimal stress group, there were no clear differences between the groups (P=0.5, $F_{3,54.4}=0.84$, and $\eta_{p}^{2}=0.04$), and no differences were found from any PFC quads.

### 3.6 Effects of Stroke History

We examined the effect of stroke history (SCDhs1 versus SCDhs0 versus healthy controls) using the analysis model described previously. The model showed a significant interaction with the Stroop-induced elevated stress, requiring two separate analyses stratified by the first task performed. However, due to the limited number of SCDhs1 subjects in the N-back first group (n=2), we did not perform a statistical test on the effect of stroke in the minimal stress group.

**Fig. 10:**
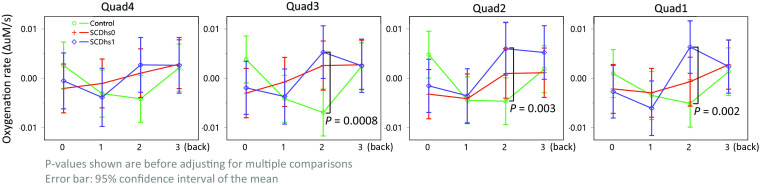
Oxygenation to N-back in SCD with a history of stroke was even greater compared to healthy controls. Oxygenation responses during 2-back in quads 1 to 3 showed significantly greater oxygenation compared to controls. There were no clear differences between SCDhs0 and SCDhs1.

In the elevated stress group (i.e., Stroop first), which consisted of a sufficient number of SCDhs1 subjects and was balanced (SCDhs1 = 6, SCDhs0 = 7, and control = 8), we observed a similar contrast as in the SCD versus control. And there was an even greater spread between SCDhs1 and controls during 2-back ($P_{\text{all quads}}=0.001$, $t_{55.04}=3.39$, and d=0.46). Significant differences were found in quad 1 (P=0.002, $t_{100.1}=3.21$, and d=0.32), quad 2 (P=0.003, $t_{98.21}=3.01$, and d=0.30), and quad 3 (P=0.0008, $t_{98.3}=3.46$, and d=0.35) (Fig. 10). Meanwhile, we did not detect clear differences between SCDhs1 and SCDhs0.

## 4 Discussion

### 4.1 N-back Performance in SCD versus Healthy Controls

We investigated the impact of SCD on PFC oxygenation during an N-back working memory task, utilizing fNIRS. Our results showed that N-back accuracy decreased, and the response time increased in all participants as the N-back became more difficult. We did not find any significant difference in accuracy attributed to SCD or a history of stroke. However, there were strong tendencies toward slower response times in the SCD group, particularly in the SCD group with a history of stroke (SCDhs1) during 3-back and in the SCD group without a history of stroke (SCDhs0) during 1-back compared to controls (P=0.02, 0.025, and 0.007, respectively).

### 4.2 PFC Utilization in SCD

All participants’ grand average PFC oxygenation response showed a monotonically increased oxygenation rate with increasing N-back difficulty. The following group analysis found a greater oxygenation increase in the SCDhs1 group compared to the healthy control group during 2-back. These findings suggest the potential effects of SCD and stroke on short-term memory processing. However, these observations interacted with the elevated stress in half of the participants, which made it difficult to draw a definitive conclusion.

### 4.3 Model-Based fNIRS Signal Filtering Confounders

One notable contribution of this work is the utilization of a model-based filtering method to refine fNIRS measurements. This technique allowed for the correction of confounding influences from non-neuronal but rather physiological origins, such as skin blood flow or breathing patterns. We reduced the confounding influences due to skin blood flow using finger PPGa, a representation of peripheral blood flow changes, as an input to a dynamic systems model based on the assumption that we could recover scalp blood flow influences through modeling. This was evident in 3-back results from Figs. 7(b) and 7(c), even for the HbR signal that is typically less contaminated, highlighting its effectiveness in the absence of short-separation scalp-blood measurements. The similarity between finger PPGa and another short-separation NIRS device measuring scalp blood flow suggests that scalp blood flow and finger PPGa have a common origin, and there are additional factors to model (Fig. S2 in the Supplementary Material). As a result, we obtained more characteristic responses to the N-back task, demonstrating the effectiveness of our model-based filtering [Figs. 7(b) and 7(c)]. Although we have demonstrated the utility and potential of the model-based filtering method in this particular study, extending this technique to other fNIRS datasets in general would require validation against standard approaches, such as short-channel regression or general linear modeling with systemic physiological regressors.

### 4.4 Robust Quantification of PFC Oxygenation to N-back

To quantify PFC oxygenation responses to mental tasks, we used the slope of oxygenation changes beginning 10 s after each trial. We chose this approach because it provided a less biased quantification of fNIRS oxygenation as it showed a monotonic increase toward difficult tasks, compared to other metrics that we tried, such as signal mean, time-to-peak, or total amplitude of oxygenation changes. These other metrics can be biased by signal spikes caused by motion artifacts and/or the rapid transient period caused by physiological changes during the first 10 s of each trial. These considerations provided a fail-safe mechanism, ensuring that our final results were robust to variations in signal cleaning and preprocessing procedures.

### 4.5 Longer Response Time in SCD and What it Means

Our study found a potential effect of SCD and stroke on short-term memory processing in the behavioral results, as evidenced by longer response time in certain N-back tasks. Specifically, participants with SCD with a history of stroke (SCDhs1) exhibited longer response times in 3-back tasks, and SCD with no history of stroke (SCDhs0) showed longer response times in 1-back tasks compared to healthy controls. These longer response times may reflect slower and less efficient processing speed, potentially attributable to white matter loss in SCD that can cause delays in axonal transmission.^18^ Our findings align with previous studies that reported longer response times in SCD during cognitive tasks.^30^^,^^54^

### 4.6 PFC Oxygenation to N-back Interacted with Elevated Stress

There was an interaction between the elevated stress due to Stroop and PFC oxygenation trends to N-back, particularly in the healthy control group. This interaction made it challenging to analyze the effect of SCD and stroke with confidence. Our data showed significantly greater oxygenation in SCD than controls, only when a Stroop test was administered prior to N-back. Correspondingly, this also implied significantly lower oxygenation responses in healthy controls compared with SCD when the Stroop test was administered first. Healthy controls exhibited negative PFC oxygenation during the “difficult,” 2-back tasks and positive PFC oxygenation during the “easy,” 0-back tasks, which were not typical. This atypical pattern contributed to a significant contrast in SCD and healthy controls. Such atypical responses were not observed in the SCD group, even though they performed Stroop prior to N-back. This interaction with elevated stress set by Stroop was significant.

The color-word incongruent/conflicting Stroop test is known to activate the executive function of the brain, which is associated with the lateral PFC;^55^^,^^56^ it requires participants to suppress the automatic response of reading the word and answer the color of the calligraphy of the word. Studies have shown that the Stroop test also activates sympathetic-driven peripheral vasoconstriction.^32^^,^^57^ These activities from both the autonomic and central nervous systems could cause mental stress. Furthermore, such stress in our subjects might have been escalated due to sitting in the same place for an extended period (>30  min) until conducting the N-back as the last task of the study. It raises intriguing questions of whether or how the PFC cognitive activities differ under elevated stress levels, particularly after being exposed to stimuli that can trigger pain crises in SCD.

### 4.7 Significance of Our Study Findings

To the best of our knowledge, there has been no prior report investigating PFC oxygenation responses in SCD using fNIRS. In our study, we observed significantly greater PFC oxygenation during an N-back task in SCD patients compared to healthy controls, particularly after exposure to mental stress. This trend was consistently seen in Figs. 8, 9(b), and 10. *Post hoc* analysis further revealed that, during the 2-back task, SCD patients (including those with a history of stroke) exhibited greater oxygenation increase in PFC quads 1 to 3 [Figs. 9(b) and 10]. Given these findings and results, it is possible that SCD patients experience greater processing demands and more widespread recruitment during challenging tasks compared to the healthy controls.

### 4.8 Study Limitations

Our study has several limitations worth noting. The elevated stress induced in half of our participants showed an interaction with PFC responses to N-back. We accounted for this interaction by stratifying the analysis based on which task was performed first. Nevertheless, the effect of elevated stress on cognitive function can be complex and difficult to interpret. The previously mentioned, atypically low oxygenation response to difficult tasks in healthy controls within the elevated stress group is that example. Therefore, caution should be exercised when drawing conclusions about the effects of SCD and stroke on short-term memory processing from our results. Future studies should avoid stacking different types of mental tasks as their effects can interact and reduce statistical power. Despite this, our study found significant results when acknowledging preconditioned or elevated mental stress, which could be an interesting topic for further investigation.

From a signal processing viewpoint, the CBSI method, which relies on the linear combination of HbO (prone to non-neuronal influences) and HbR (with low SNR), operates under assumptions (i.e., the positive correlation between HbO and HbR is artifactual and a fixed ratio of HbO to HbR) that, if violated, could undermine its validity. Also the absence of short-separation channels poses challenges in isolating true neuronal components. Despite these challenges, we addressed them with motion artifact removal, bandpass filters, model-based physiological contamination filtering, and utilizing linear slope fitting for quantifying oxygenation responses. Another methodological limitation, as mentioned above (in “model-based fNIRS signal filtering confounders”), is that the model-based filtering technique introduced in this study is relatively novel and requires further validation for application to other fNIRS studies. Furthermore, we grouped fNIRS channels into quadrants, which might dilute localized responses. However, we believe that this strategy offered robustness given different head sizes across subjects and signal processing challenges.

Another limitation is the small sample size, particularly in SCD patients with histories of stroke, consisting of both silent and overt stroke phenotypes. These two phenotypes can have different brain physiology and hemodynamics, influencing the fNIRS measurements. Additionally, we did not find consistent correlations between response time, accuracy, and oxygenation trends, making it difficult to describe what cognitive decline means using these primary metrics. For example, response time and accuracy did not consistently correlate, nor did changes in oxygenation consistently indicate better or worse behavioral performance. Despite these limitations, our study provides an examination of PFC oxygenation during short-term memory tasks in SCD patients. This research contributes to a better understanding of the cognitive decline reported in SCD and highlights the need for more extensive studies with more robust experimental designs to uncover the underlying mechanisms.

## 5 Conclusion

We monitored PFC hemodynamics in SCD patients and healthy control subjects during N-back working memory tasks using fNIRS. Unwanted influences from extracerebral origins were minimized using dynamic systems modeling followed by a correlation-based signal improvement method. Our results showed a monotonic increase in average PFC oxygenation, quantified by the linear slope, as task difficulty increased in all participants. Notably, we observed greater oxygenation during 2-back tasks in SCD patients compared to healthy controls when the stress levels were elevated. However, task accuracy was not different between the groups. Also considering the longer response times in the SCD group compared to healthy controls, our findings suggest potential alterations or inefficiencies in PFC processes in SCD patients, and such changes may serve as indicators of their cognitive decline. Further investigation addressing our study limitations and the application of an fNIRS-based assessment on a larger SCD population will help monitor and understand the process of cognitive decline in SCD.

## Code, Data, and Materials Availability

The main software functions used for model-based filtering were adopted from LYSIS,^58^ and those for signal cleaning were adopted from Homer3,^40^ both of which are available free of charge. The comprehensive set of customized software codes and acquired subject data that support the findings of this article are not publicly available due to privacy concerns. However, they can be requested from the authors at jsunwoo@mgh.harvard.edu and khoo@usc.edu.
