# Supplementary material for: Functional near-infrared spectroscopy-based prefrontal cortex oxygenation during working memory tasks in sickle cell disease
Source: Neurophotonics. 2023 Oct 17;10(4):045004. doi: 10.1117/1.NPh.10.4.045004 (PMC10581024; doi:10.1117/1.NPh.10.4.045004)
Supplement: Supplementary file 1 [file NPh_010_045004_SD001.pdf]

Supplementary

PFC oxygenation and extracerebral responses to N-back

A representative example showing brain HbO, finger PPGa, and ETCO2 changes during the baseline and during an N-back task is shown in **Figure S1**. The bars on the first row indicate the difficulty levels (0-3 backs), and fNIRS channels 2 and 7 are shown as examples of lateral and medial PFC oxygenation responses to N-back. The last two rows show skin vasoconstriction and ETCO2 fluctuation changes, which were noticeably different after the N-back task began. There were more frequent drops in ETCO2 concentration, especially at the onset of each N-back trial, which indicated that subjects generally needed a deep sigh before or after each N-back trial to compensate for shallow breathing while concentrating during the memory task. These extracerebral responses may confound cerebral fNIRS measurements [35,58], and we aimed to identify and filter them out using a dynamic systems modeling technique.

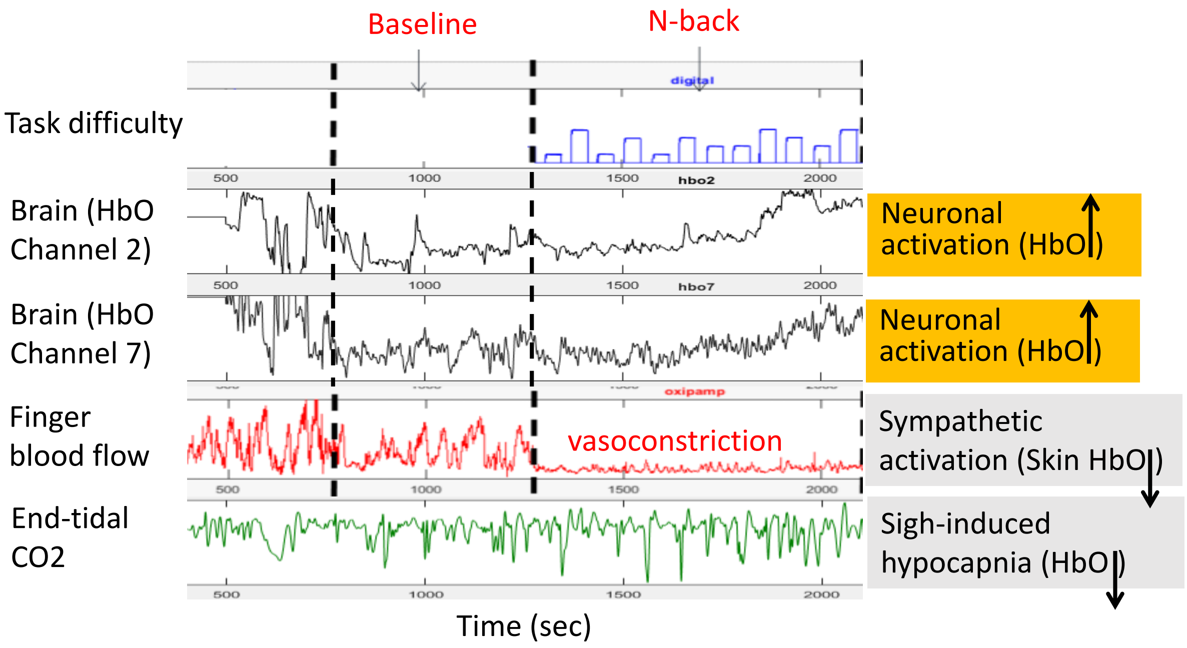

Figure S1. N-back increased brain oxygenation while possible decrease in signal amplitude due to skin vasoconstriction and hyperventilation. The signal contamination due to skin vasoconstriction and hyperventilation was estimated and subtracted from the original fNIRS HbO signal using model-based filtering.

Model-based filtering improved brain component in fNIRS: Finger PPGa served as a surrogate measure of skin blood flow changes and ETCO2 to represent breathing-induced cerebral blood flow changes.

The PPGa and ETCO2 were used as inputs to the proposed dynamic systems model, providing two advantages. First, any discrepancy between measured finger blood flow and actual scalp blood (but not measured) would be mitigated by the impulse response function found by fitting the model. Second, using physiological measurements helped avoid making blind assumptions that other methods like PCA and ICA are based on. Finger PPGa was a reasonable surrogate of scalp blood flow, and ETCO2 which reflected CO2 concentration in the brain has been reported as a critical confounder in fNIRS signal [35,47]. We also found a reasonable resemblance between the fingertip PPGa and the scalp PPGa on the forehead measured by another NIRS device equipped with a short separation (~5 mm; correlation coefficient >.5, **Figure S2**), supporting the use of fingertip PPGa as a surrogate measure of scalp blood flow influencing fNIRS measurement.

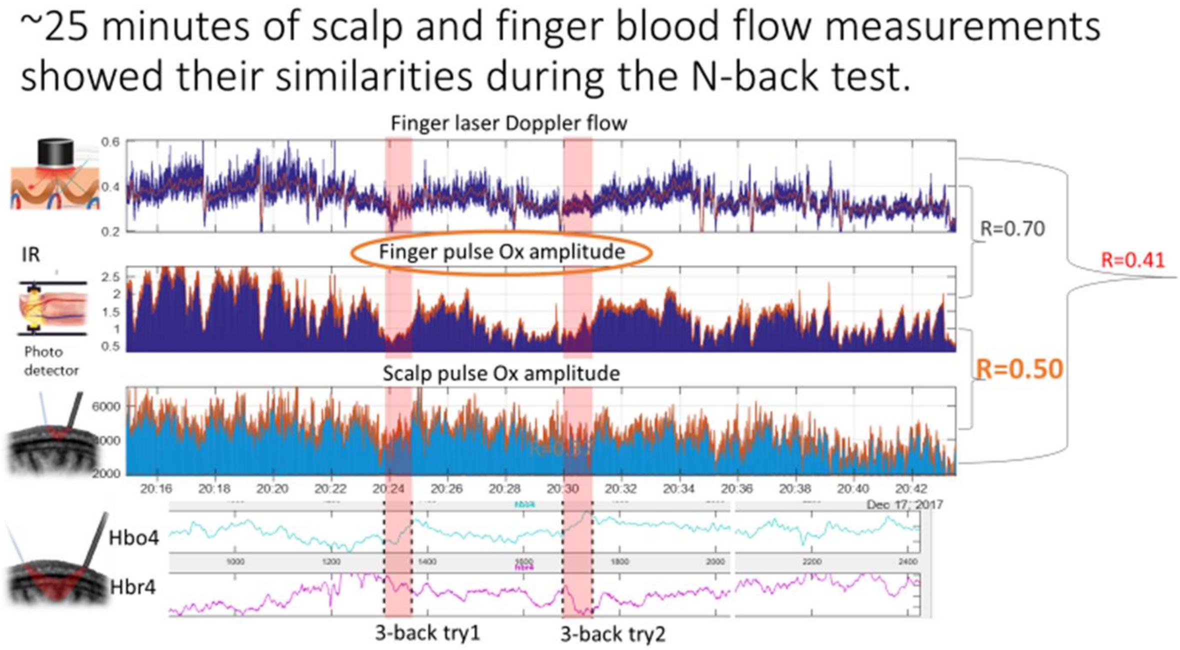

Figure S2. Scalp vs finger blood flow comparison via fNIRS, pulse ox (PPGa), and laser Doppler flowmetry showed more similar in signal waveform between the PPGa and short-separation fNIRS channel. The short-separation NIRS device was not available during the period of active enrollment.

PFC oxygenation comparison in all SCD compared to all healthy participants

We observed greater oxygenation in SCD patients during 2-back compared to healthy controls at quad 3 (*P*=.017, *d*=.16, **Figure S3**). There was also a positive and elevated oxygenation response in healthy controls during 0-back. Age and sex did not show significant effects or interactions with other variables (P_{age}=.7, P_{sex}=.9).

After we further normalized each response to its average 0-back in each channel (i.e., 1-0 back, 2-0 back, 3-0 back), there was significantly greater PFC oxygenation in SCD in all quads (*P*=.04, $F_{1,34.79}$=4.35, η_p^2=.11), and a post-hoc test revealed that SCD patients exhibited greater PFC oxygenation during 1 and 2-backs at quad 2 (*P*=.008 and .003; *d*=.26 and .30, respectively) and at quad 3 (*P*=.009 and .004; *d*=.25 and .28, respectively). In this case, the interaction from 'elevated stress' became insignificant (*P*=.07, $F_{2,73.83}$=2.76, η_p^2=.07). (Not shown; Denoted with * in **Figure S3**).

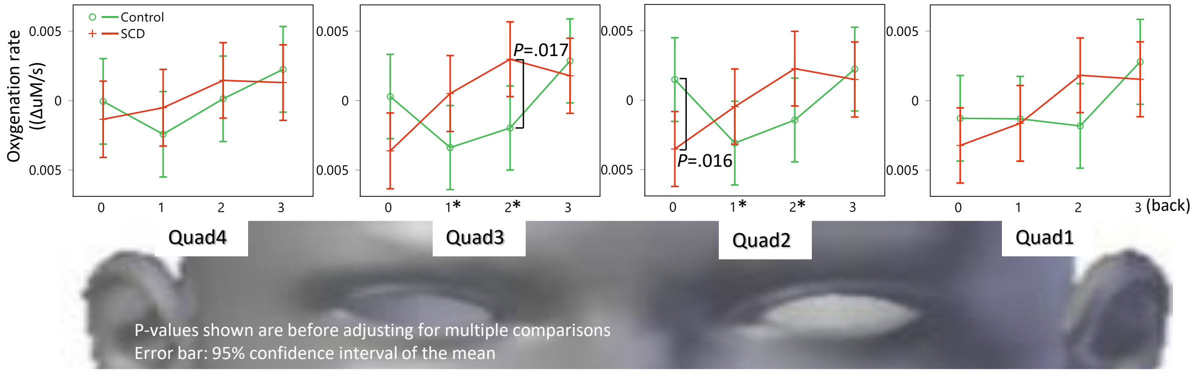

Figure S3. Oxygenation to N-back in SCD vs healthy controls indicated a difference. There was an indication of hyperactivation to 2-back in SCD in quad 3 (*P*=.017, before correcting for multiple comparisons), while there seen an elevated 0-back response in controls in general and strongest at quad 2 (*P*=.016). Age and sex did not show significance nor interaction with other variables (P_{age}=.7, P_{sex}=.9). Furthermore, N-back difficulties with the * label denoted where we found statistically significantly greater oxygenation response in SCD when they were normalized by average 0-back response of each channel (*P*=.008, .003; .009, .004, respectively from quad2:1-back, 2-back; quad3:1-back and 2-back; Not shown).

Possible differences between overt vs. silent stroke in SCD

The comparison between the overt and silent stroke in their PFC oxygenation showed significantly suppressed trends in the silent stroke SCD group compared with the overt stroke SCD group. This separation becomes even stronger when the hemoglobin count was taken into account (Not shown). There may be important/significant differences between the stroke types being overt vs silent; however, we did not analyze further, due to the limited sample size. In this section, for reference, the comparisons of all four groups are attached, where the four groups were the control (N=18), **SCDn** (no stroke; 15), **SCDhso** (Overt stroke; 3), and **SCDhss** (Silent stroke; 5) groups. Preliminary comparisons showed relatively lower accuracy rate in the SCDhss group, increased PFC oxygenation rate in SCD during N-back tasks in general, except in the SCDhss group, and longer response times in SCDhss than other groups.

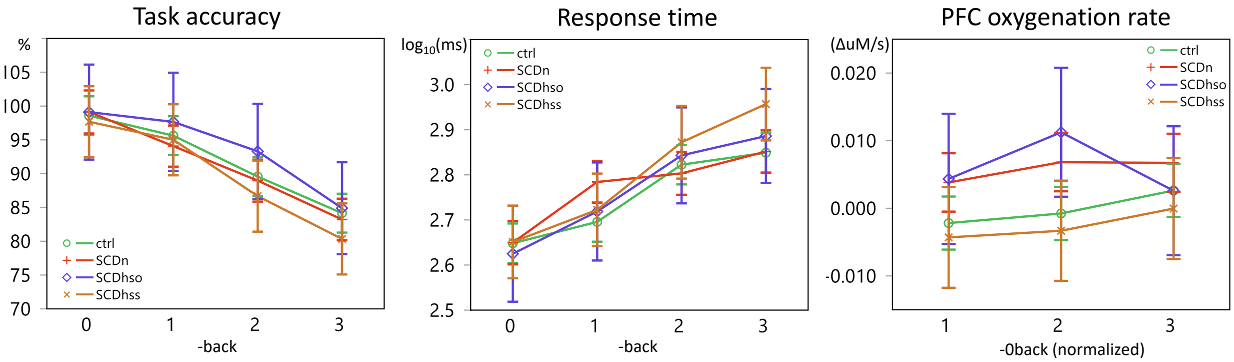

Figure S4. Possible significant differences in response time and PFC oxygenation between SCD with the histories of overt stroke vs silent stroke. Response times seemed the longest in SCD with the histories of silent stroke compared to other groups (center), while there was no clear evidence of differences in task accuracy (left). The grand average of the PFC oxygenation responses showed hyper-activation in the SCD groups, but not in SCD with the histories of silent stroke as it showed a flat response to N-back.

A representative example of the effects of each cleaning step

Motion artifact detection based on the sliding window signal variation method, followed by spline and wavelet transformation filtering, removed both step and spike types of signal artifacts and improved the quality of the fNIRS data.

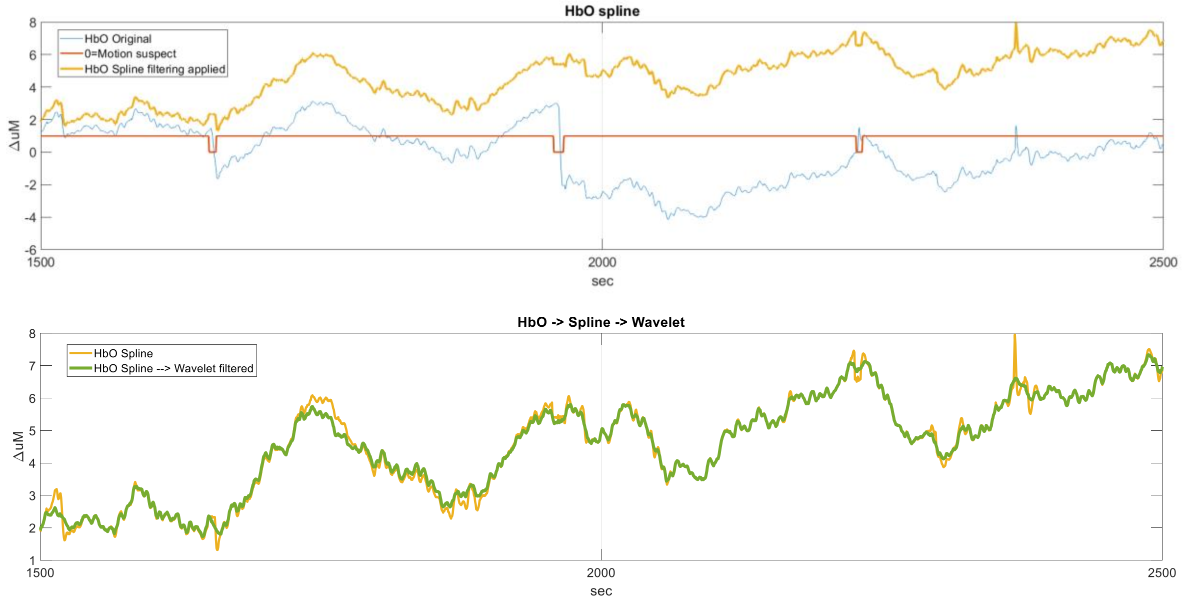

Figure S5. An fNIRS signal cleaning example of ~15 minutes excerpt: Top row, showing step and spike type signal artifacts (blue line), identified as 0 in the dark orange line, corrected by spline method shown in light orange. Bottom row shows the effect of the following wavelet filtering applied to the HbO-Spline signal, providing additional cleaning for the residual spikes.
